# Cognition, Mood and Sleep in Menopausal Transition: The Role of Menopause Hormone Therapy

**DOI:** 10.3390/medicina55100668

**Published:** 2019-10-01

**Authors:** Giulia Gava, Isabella Orsili, Stefania Alvisi, Ilaria Mancini, Renato Seracchioli, Maria Cristina Meriggiola

**Affiliations:** Gynecology and Physiopathology of Human Reproduction, S. Orsola-Malpighi Hospital, Department of Medical and Surgical Sciences (DIMEC), University of Bologna, 40138 Bologna, Italy; isabella.orsili@libero.it (I.O.); stefania.alvisi@gmail.com (S.A.); mancini.ilaria89@gmail.com (I.M.); renato.seracchioli@unibo.it (R.S.); cristina.meriggiola@unibo.it (M.C.M.)

**Keywords:** menopause, hormones, menopause hormone therapy, cognition, mood, sleep

## Abstract

During the menopausal transition, which begins four to six years before cessation of menses, middle-aged women experience a progressive change in ovarian activity and a physiologic deterioration of hypothalamic-pituitary-ovarian axis function associated with fluctuating hormone levels. During this transition, women can suffer symptoms related to menopause (such as hot flushes, sleep disturbance, mood changes, memory complaints and vaginal dryness). Neurological symptoms such as sleep disturbance, “brain fog” and mood changes are a major complaint of women transitioning menopause, with a significant impact on their quality of life, productivity and physical health. In this paper, we consider the associations between menopausal stage and/or hormone levels and sleep problems, mood and reduced cognitive performance. The role of estrogen and menopause hormone therapy (MHT) in cognitive function, sleep and mood are also discussed.

## 1. Sexual Steroids and Central Nervous System: Biologic Aspects

During their lifetime women experience dramatic fluctuations in the levels of the sexual hormones estradiol, progesterone and also androgens when going through the different stages of life, from menarche to menopause [1]. These fluctuations have a significant impact on the whole body including the central nervous system (CNS) and can be responsible for modifications in behavior, cognition and mood.

Sex steroids play a role in the brain, on both cortical and sub cortical structures, through genomic and non-genomic paths [2]. Even though most of the evidence in this field relies on non-human studies, several molecular and cellular processes are thought to be involved in determining changes in the structure and function of neural systems through both gene expression modulation and activation of signaling pathways. Sex steroids are able to modify several functions including behavior, cognition and memory, sleep, mood, pain and coordination, amongst others. They exert their function through receptors both in the nuclei and along the membranes at synapsis, spine and mitochondria. They have also been found in the glia providing regulation in myelin formation and a potential role in demyelinating diseases.

Steroid hormones active in the CNS are called neurosteroids. They may be peripherally produced steroids able to cross the blood-brain barrier or synthesized in the central and peripheral nervous system by neurons and glial cells either via de novo synthesis from cholesterol or from local metabolism of intermediate steroids produced in the periphery [3]. Even if levels of steroid hormones in peripheral blood are different from those in the CNS, measurement of their plasma levels is still important for the understanding of their role in CNS activity as they can cross the blood-brain barrier.

Different hormones can provide brain regulation in a sexually specific way: neuroprotective effects of estrogens are more evident in females than males, and androgens seem to be more active in males in the recovery from demyelinating events, while progestogens seem to be more effective in the male in the reduction of apoptosis and abnormal proliferation after a trauma or a stroke [4].

In the CNS there is a wide distribution of estrogen receptors (ERs) localized in areas of the brain involved in memory and executive function. The ER isoform, ERβ, is expressed mostly in the cerebral cortex and hippocampus, whereas ERα signaling is largely represented in magnocellular cholinergic neurons of the basal forebrain [5]. In the basal forebrain and in the hippocampus, estradiol has been demonstrated to be able to induce a trophic effect fundamental for memory and executive functions [6,7]; furthermore, estrogens mediate neurotransmitter interactions in the prefrontal cortex, which is relevant to executive functions [8]. Estrogens are also associated with an increase in dentate gyrus neurogenesis [9].

Estrogens elicit their function involving many neurotransmitter systems, such as acetylcholine, serotonin, noradrenaline and glutamate. In particular, the cholinergic neurotransmitter system is relevant in memory processes [10]. The method by which estrogen exerts its action on the brain includes neurotrophic and neuroprotective actions specifically, enhancing synaptic plasticity, neurite growth and hippocampal neurogenesis and protecting against neural injury and apoptosis [11]. Furthermore, estrogen seems to improve mitochondrial function, enhancing adenosine triphosphate (ATP) production and mitochondrial respiration, which is very important in a site with high energy requirements such as the brain [12]. Other types of estrogen action in the brain are DNA repair and promotion of an antioxidant effect [13,14]. Estrogen has also been associated with increased C-reactive protein levels (an inflammatory marker), which has been linked to impaired cognitive function.

Similar to estrogen, progesterone is also a potent regulator of neurogenesis, cell survival and bioenergetic systems. They do not have synergistic action and their co-administration leads to a lower response than with the administration of a single compound [15,16]. Progesterone acts both through the classical pathway binding to its receptors (PRα and PRβ) and regulating gene transcription and, together with allopregnanolone and dihydroprogesterone (DHP), through the non-classical pathway activating different signaling cascades and the transcription of various genes. The main effects of the two pathways are the promotion of anti-apoptotic and cell survival effects, bioenergetic regulation and a significant effect on neural cell proliferation [15]. Progesterone also exerts its action on glial cells mainly promoting oligodendrocyte proliferation and action [17,18]. Oligodendrocytes can produce progesterone and transform progesterone from the bloodstream into DHP and allopregnanolone, which regulates myelination and modulates gamma-aminobutyric acid (GABA-A) receptors [18] 

Progesterone, and especially allopregnanolone, is able to promote the GABAergic system inhibiting synaptic transmission, producing what is believed to be an anti-anxiety effect similar to that of benzodiazepines [19,20,21]. In humans, decreased levels of allopregnanolone are linked with depression and antidepressant drugs are able to determine and increase this metabolite [22]. The GABAergic role of progesterone in the hippocampus explains why exogenous administration of progestins has a negative impact on the cognitive performance of healthy women in working memory tests [23]. Progesterone and allopregnanolone are able to influence the dopaminergic systems with an observed improvement in motor sensory functions during the phases of the menstrual cycle when progesterone is higher [22]. The positive modulation of allopregnanolone on the release of dopamine can also have a possible effect on drug abuse and depression [24].

The brain is also able to locally produce the androgen dihydrotestosterone independently of the gonads [25]. Androgens in the animal model have been demonstrated to be able to induce neurogenesis and spine synapses in the hippocampus. Moreover, like estrogens, androgens have neuroprotective effects. Considering the relationship between androgens and neurotransmitters, although there are fewer studies, it seems that testosterone in particular may increase serotonergic tone (also through its conversion to estradiol) and the effect of noradrenergic anti-depressants agents. Even though much still remains to be understood, dehydroepiandrosterone (DHEA) appears to have antioxidant, neuroprotective and anti-glucocorticoid effects [26]. Through these mechanisms it can reduce anxiety and improve cognitive deficits and psychotic and depressive symptoms.

## 2. The Influence of Menopausal Transition and Menopause Hormone Therapy on Cognition

### 2.1. Menopausal Transition and Cognition

During menopausal transition many women complain of memory problems such as difficulty with words, forgetfulness and “brain fog” [27], thus suggesting that hormonal changes related to menopause may be responsible for changes in cognition. Elderly women also appear to be at greater risk than men for age-related dementia suggesting sex differences that are not fully explained by longevity [28]. 

However, in a recent cross-sectional study, women in their early midlife demonstrated better performance in detailed memory tasks when compared to age-matched men [29]. These sex differences were attenuated in the post-menopausal years. In the study by Rents et al., higher plasmatic estradiol levels were associated with better performance. Another study previously reported lower cognitive impairment associated with higher levels of estradiol in women already reporting cognitive decline [30]. Other studies did not demonstrate any relationship between cognition and cognitive problems and estrogens levels [31].

Longitudinal data considering the impact of menopausal transition on memory and cognitive function are scarce. Two longitudinal studies reported subtle decrements in cognitive function: the Kinmen Women-Health Investigation (KIWI) did not evidence significant cognitive decline, with the exception of verbal fluency, in an 18-month follow-up of pre-menopausal women [32]. The Study of Women’s Health Across the Nation (SWAN) reported an impairment of cognitive performance mostly in learning abilities during menopause transition, with subsequent improvement to pre-menopausal levels in the post-menopausal period [33]. The SWAN study has been ongoing since 1996, observing 3302 women throughout the whole menopausal transition (https://www.swanstudy.org/); in the next few years we believe that this study will provide a unique insight into the long-term effects of hormonal changes in middle-aged women. 

It should be noted that not only events occurring during menopausal transition but also lifelong hormonal processes seem to be significant for cognition. A recent observational study of 1315 women showed that a longer fertile period (later age at natural or surgical menopause) was associated with better verbal memory [34]. On the other hand, in another observational study of 3602 women, a longer fertile period was not associated with a reduced risk of dementia [35].

### 2.2. Menopause Hormone Therapy and Cognition 

#### 2.2.1. In Women without Dementia

Despite medical literature which highlight the deep connection between estrogen and cognitive function, data regarding the relationship between menopause hormone therapy (MHT) and its neuroprotective outcomes remains conflicting. Although several observational studies have demonstrated a positive effect of hormone therapy on Alzheimer’s disease (AD) [36,37,38,39,40], MHT being associated with a 29% reduction in AD in the meta-analyses of observational studies [41], large clinical trials such as the Women’s Health Initiative (WHI) and Women’s Health Initiative Memory Study (WHIMS) did not support those findings [11]. 

WHIMS was a randomized, placebo-controlled clinical trial, and the first large, long-term study to address the cognitive effects of MHT (0.625 mg of conjugated equine estrogen (CEE) plus 2.5 mg of medroxyprogesterone acetate) in the prevention of AD, in 4532 post-menopausal women (over 65 years of age) enrolled in the larger WHI study. Results showed that after a mean follow-up of 4.2 years, MHT failed to reduce general cognitive decline and was associated with a substantially and clinically important increased decline in Modified Mini-Mental State Examination (MMSE) total scores when compared with placebo [42].

Subsequent analysis of 2947 WHIMS estrogen-alone treated women (aged 65‒79) showed that mean MMSE scores were significantly lower in the estrogen group compared with placebo. The adverse effect of estrogens was more pronounced in women with lower cognitive function at baseline [43].

In the analysis of data from the Nurses’ Health Study, a prospective cohort of over 13,807 women aged 70 to 81, little difference was found in cognitive decline between current hormone users and those that had never used them. Long-term users (at least 5‒10 years of use) of estrogen or estro-progestin showed an increased risk of substantial decline in most cognitive tests. The impairment was more evident in women starting hormones at an advanced age [44]. Subsequent analysis showed that MHT users with apolipoprotein E (APOE) E4 allele presented worse cognitive impairment [45].

In 2008, a meta-analysis of clinical trials investigating MHT (estrogen or estrogen-progestin therapy) in post-menopausal women (over 60 years of age) concluded that MHT does not protect against cognitive function decline in normal women [46].

It should be observed that studies involving older women (the WHIMS was over 65 years of age) did not consider the so called “timing hypothesis”. In fact, further studies, similar to the cardiovascular risk, have suggested that estrogen can be neuroprotective only if commenced shortly after the onset of menopause [47,48]. Sustaining the “critical window hypothesis”, some epidemiologic studies have suggested that MHT in early post menopause is linked with a lower risk of dementia, whereas its later use is not [38,39,49,50]. 

Data from the Cache County Study revealed that women using MHT had a reduced risk of AD compared with non-MHT users only if treatment was started soon after menopause (within five years) [38,39]. Similarly, Whitmer et al. showed that among 5504 post-menopausal women, MHT in midlife was associated with a protective effect against cognitive impairment whereas starting MHT later in life could have a negative effect [49]. Other studies did not confirm that MHT use close to the time of menopause was clearly associated to better cognitive performance later in life or to a reduced risk of dementia [51,52]. 

There have also been other limited clinical trials regarding early MHT and cognitive performance later in life. The WHIMS‒Young (WHIMSY) study examined 326 post-menopausal women between 50 to 55 years of age randomized for MHT (CEE with or without 2.5 mg medroxyprogesterone acetate over a mean of 7.0 years) or placebo. CEE based therapies produced no overall sustained benefit or risk to cognitive function when administered to post-menopausal women aged 50 to 55 years. Cognitive evaluation performed up to 67 years of age did not show sustained benefits of MHT over placebo [53]. Even though they do not support the theory of cognitive improvement in MHT users, these studies provide reassurance that hormone therapy does not adversely influence cognition in young post-menopausal women.

#### 2.2.2. In Women with Dementia

Cognitive decline and dementia are a growing public health problem. The inability to recall information, defined as impaired episodic memory, is a potentially alarming symptom of the very early signs of AD or other forms of dementia. AD is the most common cause of dementia and emerges more frequently in women than in men, with some authors hinting at sex-specific differences in the incidence of AD [54]. This condition is characterized by a progressive loss of episodic memory and cognitive function, subsequently causing language and visuospatial impairment, which are often accompanied by behavioral disorders such as apathy, aggressiveness and depression. 

Considering the significance of this disease, research in this field has attracted considerable scientific and public interest. The role of estrogen has been assessed not only in the maintenance of cognitive function in non-demented women but also in the prevention and/or treatment of AD. 

In vivo and in vitro preliminary data suggest that estrogen may play a role in the prevention of amyloid deposition. Estradiol has been shown to attenuate tau hyperphosphorylation [55] and deposition of amyloid b [56] and has also been shown to improve the inflammatory sequelae of amyloid b [57]. 

In the Cache County Study, a prospective study of incident dementia among 1357 men (mean age 73.2 years) and 1889 women (mean age 74.5 years), women using MHT had a reduced risk of AD when compared with non-MHT users (adjusted HR 0.59), whereas there were no apparent benefits with current MHT use unless they had been used for in excess of 10 years [38].

Other large epidemiologic studies have addressed the role of exogenous estrogens and the risk of dementia. In 2001, the first meta-analysis of these observational studies reported a reduced risk of dementia in MHT users, even with some methodological limits of the studies [58], whereas subsequent meta-analysis of prospective studies failed to show any association between MHT and all-cause dementia and AD [59]. 

Recently, the Kronos Early Estrogen Prevention Study (KEEPS) showed that transdermal 17-beta estradiol therapy was linked to a decrease in amyloid-beta deposition on neuroimaging studies, particularly in apolipoprotein E (APOE) E4 carriers [60].

More recently, data provided by Savolainen-Peltonen et al. showed that systemic hormone therapy users have a higher risk of AD when compared to vaginal estradiol users and that long term use of systemic hormone therapy could be burdened by a globally increased risk of AD, independent from the age at initiation of systemic hormone therapy [54]. The risk of AD was similar between estradiol-only users and oestrogen-progestogen users, and in oestrogen-progestogen users, the risk was not associated to specific progestogens (norethisterone acetate, medroxyprogesterone acetate, or other progestogens). It should be noted that for shorter treatment the risk of AD was not increased among those who had commenced either estrogen therapy or estrogen plus progestin therapy before the age of 60.

Several small randomized clinical trials have considered the possibility of the treatment of women with Alzheimer’s with estrogen. In the largest study of 120 women, estrogen administration for one year (conjugated equine estrogens 0.625 mg or 1.25 mg vs placebo) did not slow progression of the disease nor did it improve global, cognitive or functional outcomes [61]. Subsequent meta-analysis of seven trials in reported similar results [62]. 

We believe that those studies should not modify our current clinical practice and decision making regarding MHT. In fact, even if some observational studies had inconsistent and mixed results, it should be remembered that several randomized clinical trials supported the safety profile of MHT on cognition in early menopause.

Overall, young menopausal women without contraindication to MHT and with impaired quality of life because of night sweats, vasomotor symptoms or disrupted sleep can benefit from MHT with several studies providing reassurance that MHT does not adversely affect cognition in these women. On the counterpart, it should be noted that, consistently with guidelines, MHT should not be prescribed with the sole purpose of AD or memory loss prevention in women without these symptoms or in those not willing to start hormonal treatment.

Despite the amount of data already available on this topic, future research should focus on long-term risks of MHT on cognition and AD and on the different effects on cognitive outcomes of various type of molecules.

## 3. The Influence of Menopausal Transition and Menopause Hormone Therapy on Mood

### 3.1. Mood Disorders during Menopausal Transition 

During menopausal transition, women are at higher risk of developing depression, stress, anxiety and emotional distress [63]. Several researchers have attempted to examine the reasons why women are more likely to experience greater susceptibility to depression at certain stages throughout their lifetime. More specifically, we can describe precise periods of biological vulnerability in women’s lives such as the phases of the menstrual cycle, pregnancy and postpartum, and menopausal transition. There are some windows of vulnerability for depression, or reproductive-related depressive episodes, such as increased sensitivity experienced by some women to changes in hormonal levels that characterize the luteal phase of the cycles, the postpartum period and menopause transition [64]. Depressed mood and sleep problems (insomnia, nighttime awakening or waking early) are likely to be mutually related and must be treated specifically [65].

Longitudinal studies show that there are many other factors that may have a significant influence on mood levels. In particular, these include demographic, psychosocial and health related characteristics such as unemployment, low level of education, being Black, Japanese or Hispanic, poor social support, smoking, chronic medical diseases, a history of anxiety, history of postpartum depressive symptoms, nulliparity, VMS, stressful life events, death of the partner, body mass index (BMI) and self-esteem. Body dissatisfaction may be common in midlife women [66]. There is evidence that age and BMI are positively related to the intensity of menopause symptoms, as well as changes in lifestyle factors (e.g., physical activity), which in turn can have an influence on women’s mood [67]. Self-esteem is directly associated with depression and anxiety [68]. In particular, low self-esteem can predict the occurrence of distress in relation to symptoms of menopause, such as hot flashes, and may be related to women’s health as well as perceived stress and mood during this period of their life [69].

Some studies have examined the association between depression and hysterectomy with or without oophorectomy, obtaining mixed results [70,71,72,73]. A study by the Australian Longitudinal Study on Women’s Health, which enrolled 5336 women, detected an elevated risk of depressive symptoms, 20% higher in women with hysterectomy and ovarian conservation and 44% higher in women with hysterectomy without ovarian conservation compared to controls (women without hysterectomy) [74].

Considering the causes of mood alteration during menopausal transition, the role of estrogens should be considered, even if the link of estradiol (either declining levels or low levels) in the precipitation of peri-menopausal depression (PMD) is not yet fully understood [75]. Estradiol (E2) regulates the synthesis, metabolism and receptor activity of the classical neurotransmitters implicated in depression (serotonin, dopamine and norepinephrine) [76]. Researchers have demonstrated the presence of a wide distribution of estrogen receptors in the brain. Estrogen activity found in regions known to be involved in mood and cognitive regulation (e.g., prefrontal cortex, hippocampus) is contributing evidence of the concept of mediating effects (and possible therapeutic effects) of this hormone on mood. In general, the effects of E2 on serotonin (5-hydroxytryptamine (5-HT) may be described as favorable to mood, with an increase in 5-HT synthesis and availability [64]. In particular, a reduction of monoamine oxidases (MAOs) A and B activity after E2 administration has been documented, with a limitation of 5-HT degradation as a result [77]. This also promotes the increase of both isoforms of tryptophan hydroxylase, the rate limiting enzyme of serotonin production [77]. Estrogens also improve mitochondrial respiratory efficiency, helping to prevent the formation of oxygen free radicals that are known to negatively affect mitochondria energetics in depression. Estrogen effects also promote noradrenaline (NE) availability through a reduction of monoamine oxidases (MAOs) and an increase in the activity of tyrosine hydroxylase, the rate limiting enzyme in the synthesis of catecholamine [78]. Acute E2 administration stimulates dopamine b-hydroxylase (DBH) gene transcription, catalyzing the hydroxylation of dopamine to form NE [64]. Finally, estrogen may also play a role as an anti-depressant, because of its stimulating effect on then brain-derived neurotrophic factor (BDNF), an important neuroprotective and growth factor agent, found to be deficient in depression [76].

In comparison with cross-sectional studies, longitudinal studies are a more suitable way for the assessment of the potential association between menopause transition stage and depression, as well as in the direction of association [79]. The SWAN aimed to examine the course of clinical depression during a 13-year follow-up period. The results from their study showed that about 30% of those who developed depression during the course of the follow up experienced a worsening of persistency or recurrence of depressed mood. The finding was confirmed also among those with new onset cases of depression [80]. Likewise, a parallel tendency of the increased risk for depressive symptoms in menopausal transition has been observed in other large, longitudinal studies such as the Harvard Study of Midlife Mood and Cycles [81], the Australian Longitudinal Study of Women’s Health (ASWH) [82], and the Seattle Midlife Women’s Health Study [83]. The Penn Ovarian Aging Study (POAS) hypothesis, however, was that the risk of higher depressive symptoms increased in the years before menopause and decreased in the years after menopause, in relation to the final menstrual period (FMP). The study evaluated 203 pre-menopausal women who had reached natural menopause during a 14-year follow-up. The risk of depressive symptoms was significantly higher in women in menopause transition, before FMP with a lower risk after FMP [84]. 

The existence of menopause-associated depression has been a controversial topic among clinicians, despite the undeniable evidence of a heightened increase of major depressive disorder (MDD) during menopausal transition. However, it is not possible to define an “estradiol withdrawal hypothesis”, since several hormonal and metabolic factors could confound the effects of estradiol withdrawal in peri-menopausal women. Furthermore, conflicting methodologies used to characterize reproductive staging or assess psychiatric conditions in various studies are confounding factors to the matter in question. Methodologic differences of note include menopausal state (peri-menopause vs. post-menopause), determination of state (earlier studies have used age as a proxy measure), baseline symptomatology (asymptomatic vs. depressive symptoms vs. syndromic depression), route of hormone administration (transdermal vs. oral), and symptom or syndrome measurement. For these reasons much of the literature on women’s mental health looks at the transition to menopause from a disease-risk point of view [63]. According to this conception, Gordon et al. underlined how it is not the absolute levels of hormones that make the difference to the onset of MDD, but the individual variability and sensitivity to reproductive hormonal change [85].

### 3.2. Menopause Hormone Therapy and Mood

#### 3.2.1. In Non-Depressed Women

In peri-menopausal women, one randomized controlled trial assessed mood in 83 non-depressed peri-menopausal women during six months of hormone therapy with CEE for two weeks followed by two weeks of CEE plus medroxyprogesterone acetate (MPA). No significant effects on mood were observed [86]. Recently another study assessed 38 non-depressed peri-menopausal women and reported no significant changes in mood [87].

The role of estrogen administration in the prevention of depressive symptoms in peri-menopausal and early post-menopausal transition has been assessed in one randomized trial. One hundred and seventy-two women were randomized for transdermal E2 (0.1 mg/d) plus intermittent oral micronized progesterone (200 mg/d for 12 days every three months) or placebo. After one year, women receiving estrogens plus progesterone were significantly less likely to present depressive symptoms compared with women receiving a placebo (32.3% vs. 17.3%), this effect being more evident in women in the early peri-menopausal stage [85].

Considering post-menopausal non-depressed women, mood effects of hormone therapy have been assessed in several studies showing a lack of significant effects of estrogens on mood when used in non-depressed post-menopausal women. Haines et al. failed to recognize any effect on mood after one year of treatment with oral estradiol (1 or 2 mg) versus a placebo [88]; similarly, Girdler et al. did not report any significant difference in mood in users of MHT (CEE 0.625 mg/d or CEE 1 MPA, 10 mg/d) versus a placebo [89]. The study by Almeida et al. also reported no significant changes in mood symptoms after 20 weeks of treatment with oral E2 versus placebo [90]. Another large trial on 412 post-menopausal women (mean age 71 years) confirmed the absence of significant effects of hormone therapy on mood symptoms in healthy women [91].

While the KEEPS study reported a positive impact of oral CEE versus transdermal E2, the Heart and Estrogen/Progestin Replacement Study (HERS), the Women’s Health Initiative Study of Cognitive Aging (WHISCA) and the Women’s International Study of long Duration Estrogen after the Menopause (WISDOM) did not report a significant impact of MHT on mood. 

For this reason, MHT should not be proposed to non-depressed, asymptomatic peri-menopausal women to prevent or alleviate mood symptoms. Estrogen seems to have a potential role among specific sub-populations at risk of depressive symptoms during menopausal transition. 

#### 3.2.2. In Depressed Women

There is some evidence that hormonal therapy has potential anti-depressant effect similar to that of classic anti-depressant agents when administered to depressed peri-menopausal women [79]. Even though there are the aforementioned E2 antidepressant properties, the acceptability of estrogen therapy (ET) as part of the therapeutic source for depression remains restricted. A recent review from Soares et al. attempted to classify the efficacy of estrogen-based interventions for depression, shedding light on the diverse clinical effects of MHT on mood on the basis of its administration in peri-menopausal or post-menopausal women and in particular if these women had already been suffering from depressive disorders [64].

Previously reported data from Gordon et al. underline the “critical timing hypothesis,” which suggests that the beneficial effects of estradiol are observed only if it is administered proximate to the cessation of ovarian activity. Estrogen therapy is ineffective as a treatment for depressive disorders in post-menopausal women [79].

Other findings from trials enrolling recently post-menopausal women with depression and several large observational studies, including women initiating MHT shortly after menopause, suggest that menopausal hormone therapy has either a neutral or beneficial effect on mood [92]. Four small studies, including two randomized controlled trials which demonstrated the efficacy of E2 for the management of depressive disorders during peri-menopause [93,94] and other randomized controlled trials which assessed the use of MHT (estradiol valerate 2 mg plus dienogest 2 mg, estradiol 1 mg/day plus medroxyprogesterone 10 mg/day for 2 weeks and transdermal 17 -estradiol 0.05 mg/day) in post-menopausal women with depressive disorders demonstrated some improvements or no mood changes [95,96,97].

Schmidt and colleagues attempted to determine the existence of a diverse response to estradiol withdrawal in women with past pre-menopausal depression (PMD) compared with those without past depression [75]. The study contemplated three weeks of open-label administration of transdermal E2 (100 mg/d). After this period of time, participants were randomized to receive either E2 or matched placebo skin patches for three additional weeks in a double-blind fashion. In this randomized, double-blind, placebo-controlled study the authors observed that women with past PMD, who were previously responsive to hormone therapy, had a greater risk of recurrence of depressive symptoms during blind hormone withdrawal than those who remained on E2 therapy who continued to be asymptomatic, indicating that normal changes in ovarian estradiol secretion may trigger a dysfunctional behavioral state in these susceptible women. 

In conclusion, as suggested by the recent consensus recommendation by Maki et al., although estrogen may increase the response to anti-depressants in midlife and older women, their use should still be suggested when indicated for other concurrent conditions such as vasomotor symptoms [79]. Estrogen is not approved by the Food and Drugs Administration (FDA) for mood disturbance.

Although only a small proportion of women experience depressed mood in relation to menopause, it is mandatory to accurately investigate the origin of depressive symptoms in order to detect those women who had history of depression before the menopause transition and those who had the onset during the menopausal transition. Antidepressants remain the first-line treatment of depression for patients with previous history of depression [64]. In women seeking improvement of mild mood-disorder symptoms, clinicians should consider both non-pharmacologic (i.e., exercise, balanced diet and dietary supplements) and/or hormonal strategies [98].

Future research is needed to better understand the relationship between hormonal fluctuations and perimenopausal depression and the mechanisms of the windows of vulnerability across certain phases of women’s reproductive life

## 4. The Influence of Menopausal Transition and Menopause Hormone Therapy on Sleep

### 4.1. Sleep Changes Associated with Menopausal Transition

Women transitioning menopause typically complain of poor sleep quality, insufficient sleep, nocturnal awakenings and apnea. Sleep difficulties often begin during menopausal transition and their prevalence increases in post-menopausal life with rates of self-reported sleep problems ranging between 40% and 56%, compared to pre-menopausal women in their late reproductive stage, reporting rates of 31% [99]. Sleep deprivation is a known risk factor for cardiovascular disease, diabetes, obesity and neurobehavioral dysfunction [100] and can lead to increased health care costs and reduction of quality of life and work performance. Sleep disturbances can be classified into three groups: trouble falling asleep, waking up several times and waking up earlier. Longitudinal analysis of eight years of data from more than 3000 women in the Study of Women’s Health Across the Nation showed that waking during the night was the most common type of sleep problem [101].

Kravitz et al. investigated the associations between E2, follicle stimulating hormone (FSH) and sleep disturbances. Their results indicated that only changes in hormone levels, but not baseline levels, are associated with sleep disturbances. In particular, serum E2 reduction correlated with both trouble falling asleep and staying asleep, whereas increments in serum FSH levels were associated with reports of difficulty staying asleep [102]. Sowers et al. [103], however, demonstrated that women who had a slower rate of FSH change had significantly lower sleep efficiency, and in contrast with associations previously observed between changes in FSH and sleep, sleep measurements did not reflect changing levels of E2. A higher baseline E2 was associated with moderately decreased sleep quality.

Surgical menopause is associated with more severe sleep disturbances than with natural menopause [101]. Indeed, women who undergo bilateral oophorectomy are at higher risk of more severe hot flashes than women in physiological menopause [104].

Menopausal transition is accompanied by further typical risk factors for sleep disturbances other than by estrogen withdrawal itself. Vasomotor symptoms are one of the most characteristic manifestations of the climacteric, and many studies have reported a strong association between sleep disorders and vasomotor symptoms. Vasomotor symptoms arise with estrogen decrease through a complex mechanism of actions, more complex than just estrogen withdrawal, involving central noradrenergic activity, as well as serotonergic mechanisms [105], and, most recently, involvement of hypothalamic kisspeptin, neurokinin B and dynorphin (KNDy) neurons [106]. Longitudinal data from the SWAN study found that women with moderate to severe hot flashes (6–14 days in a two-week period) are almost three times more likely to suffer from frequent nocturnal awakenings compared to women without hot flashes [102]. 

Many other factors associated with the aging process can be involved in the onset of sleep problems during menopausal transition such as obesity, cardiovascular disease, gastrointestinal problems, urinary problems, endocrine problems, chronic pain syndromes, the use of neuroactive medications, smoking, caffeine, selective serotonin reuptake inhibitors, bronchodilators, antiepileptic medications, thyroid hormone preparations, among others [107]. Moreover, the aging process is characterized by changes in the sleep electroencephalogram (EEG) and in sleep related-hormone secretion [108]. These findings correlate with a decline in sleep continuity, a decrease in slow wave sleep (SWS) and an increase in nocturnal wakefulness [109].

Other factors related to sleep troubles are restless leg syndrome (RLS) and obstructive sleep apnea (OSA). RLS is a common condition of the nervous system that causes an overwhelming irresistible urge to move the legs, occurring during the evening or at bedtime. Its prevalence is higher among women; however, the precise cause is still unknown. A number of theories have been proposed that include iron deficiency secondary to pregnancy or persistently high levels of estrogen during pregnancy or the decrease of estrogen and melatonin at menopause [107]. Concerning the role of estrogen in the etiopathogenesis of RLS, Saltzman et al. suggest that estrogen influences the dopaminergic system [110] and the catabolism of dopamine by inhibitory influence on the catechol-O-methyltransferase (COMT) enzyme [111]. However, one recent study found no difference between sleep difficulties across females and males, despite a higher prevalence of RLS among women [112]. Menopausal women previously suffering from RLS describe a worsening of severity after menopause regardless of the use of menopause hormone therapy (MHT) [113].

Between 47% and 67% of post-menopausal women have been found to suffer from OSA [114,115]. Physiological factors explaining this higher prevalence include higher BMI, larger neck circumference, higher waist-hip ratio and fat distribution changes, with an increase of central obesity. OSA causes a condition of apnea and hypopnea during the night which results in disrupted sleep patterns and a worsening of sleep quality, due to intermittent hypoxemia, arousals, and both rapid eyes movements (REM) sleep and SWS reduction. These arousals rarely result in complete awakening but can have a significant negative effect on the restorative quality of sleep. Patients can often suffer from excessive daytime sleepiness and insomnia or symptoms such as snoring, gasping, choking and memory impairment. 

### 4.2. Menopause Hormone Therapy and Sleep

MHT composed of estrogen alone for women who have undergone a hysterectomy and combined with progestin for women with an intact uterus is the most effective treatment of menopausal symptoms (i.e., hot flashes and night sweats) and their potential consequences (i.e., diminished sleep quality, irritability and reduced quality of life) [116]. Several studies in literature have attempted to examine the effects of MHT on sleep, although investigations are mixed and difficult to compare given the heterogeneity in study populations, methods for evaluating sleep, lack of uniformity in the definition of diagnosis of sleep disturbances and variations in MHT preparations (formulation, dose and type of administration).

In most studies enhanced sleep quality resulted from an improvement in vasomotor symptoms [117,118,119,120]. A 2015 literature review conducted by Attarian and colleagues detected that MHT administered in the form of low-dose estrogen or progestogen could improve chronic insomnia in menopausal women, with 14 of the 23 studies reviewed showing positive results [121]. There is conflicting data about the link between vasomotor symptoms at menopause and objective polysomnographic measures of sleep [121]. Finally, a study conducted by Schuessler et al. focused on the possible sedating effects of oral progesterone, revealing a distinct decline of intermittent wakefulness after micronized progesterone compared to a placebo, without affecting daytime cognitive functions, possibly through a GABA-agonistic mechanism [122].

The etiology of menopausal sleep disorder is not fully understood. However, current literature suggests multifactorial causes. Further investigations by randomized controlled studies are needed to investigate the efficacy of these treatments on preexisting insomnia, obstructive sleep apnea and restless leg syndrome, in peri-post menopausal women. Further research is also needed to determine if self-reported sleep quality in menopause is affected by different molecules, formulations and routes of administration of MHT.

## 5. Conclusions

During menopausal transition women experience dramatic fluctuations in the levels of the sexual hormones estradiol, progesterone and also androgens, which are potentially responsible for modifications in behavior, cognition, mood and sleep. 

The following short summary provides key concepts regarding MHT in menopause and perimenopause:

- **Cognitive function and cognitive disorders**: despite the deep connection between estrogen and cognition, data regarding the relationship between hormone replacement therapy and the neuroprotective outcomes still remain conflicting. Several studies have excluded any cognitive benefits of estrogen or combined estrogen-progestin therapy in women over the age of 65 without underlying dementia. Young menopausal women without contraindication to MHT and with impaired quality of life because of night sweats, vasomotor symptoms or disrupted sleep can benefit from MHT, and in several studies, MHT does not adversely affect cognition in these women.
- **Sleep disturbances**: the etiology of menopausal sleep disorder is not fully understood, but MHT can play a role enhancing sleep quality. MHT can improve sleep, reducing night sweats, but it can also act through other mechanisms, as disturbed sleep during perimenopause can occur independently of hot flashes. Further research is needed to determine if self-reported sleep quality in menopause is affected by different molecules, formulations and routes of administration of MHT. For example, the GABAergic sedating effects of progesterone should be considered in women with sleep issues.
- **Mood and depressive symptoms**: even though some data support a potential beneficial effect of MHT on mood, it should not be proposed to non-depressed peri-menopausal women to prevent mood symptoms. Estrogens can be considered in menopausal women with other concurrent conditions such as vasomotor symptoms as they may increase the response to anti-depressants.

## References

[1] Brown J.B. (2011). Types of ovarian activity in women and their significance: The continuum (a reinterpretation of early findings). Hum. Reprod. Update.

[2] Denley M.C.S., Gatford N.J.F., Sellers K.J., Srivastava D.P. (2018). Estradiol and the Development of the Cerebral Cortex: An Unexpected Role?. Front. Mol. Neurosci..

[3] Schmidt K.L., Pradhan D.S., Shah A.H., Charlier T.D., Chin E.H., Soma K.K. (2008). Neurosteroids, immunosteroids, and the Balkanization of endocrinology. Gen. Comp. Endocrinol..

[4] Zárate S., Stevnsner T., Gredilla R. Role of Estrogen and Other Sex Hormones in Brain Aging. Neuroprotection and DNA Repair. Front Aging Neurosci (Internet) 22 December 2017. http://journal.frontiersin.org/article/10.3389/fnagi.2017.00430/full.

[5] Shughrue P., Scrimo P., Merchenthaler I. (2000). Estrogen binding and estrogen receptor characterization (ERα and ERβ) in the cholinergic neurons of the rat basal forebrain. Neuroscience.

[6] Maki P.M. (2005). Estrogen effects on the hippocampus and frontal lobes. Int. J. Fertil. Women’s Med..

[7] Tang Y., Janssen W.G., Hao J., Roberts J.A., McKay H., Lasley B., Allen P.B., Greengard P., Rapp P.R., Kordower J.H. (2004). Estrogen replacement increases spinophilin-immunoreactive spine number in the prefrontal cortex of female rhesus monkeys. Cereb. Cortex.

[8] Shanmugan S., Epperson C.N. (2014). Estrogen and the Prefrontal Cortex: Towards A New Understanding of Estrogen’s Effects on Executive Functions in the Menopause Transition. Hum. Brain Mapp..

[9] Barha C.K., Galea L.A. (2010). Influence of different estrogens on neuroplasticity and cognition in the hippocampus. Biochim. Biophys. Acta (BBA) Gen. Subj..

[10] Schliebs R., Arendt T. (2006). The significance of the cholinergic system in the brain during aging and in Alzheimer’s disease. J. Neural Transm. (Vienna).

[11] Morgan K.N., Derby C.A., Gleason C.E. (2018). Cognitive Changes with Reproductive Aging, Perimenopause, and Menopause. Obstet. Gynecol. Clin. N. Am..

[12] Zárate S., Astiz M., Magnani N., Imsen M., Merino F., Álvarez S., Reinés A., Seilicovich A. (2017). Hormone deprivation alters mitochondrial function and lipid profile in the hippocampus. J. Endocrinol..

[13] McEwen B.S. (1999). Estrogen Actions in the Central Nervous System. Endocr. Rev..

[14] Bethea C.L., Kohama S.G., Reddy A.P., Urbanski H.F. (2016). Ovarian steroids regulate gene expression in the dorsal raphe of old female macaques. Neurobiol. Aging.

[15] Irwin R.W., Yao J., Hamilton R.T., Cadenas E., Brinton R.D., Nilsen J. (2008). Progesterone and estrogen regulate oxidative metabolism in brain mitochondria. Endocrinology.

[16] Brinton R.D., Thompson R.F., Foy M.R., Baudry M., Wang J., Finch C.E., Morgan T.E., Pike C.J., Mack W.J., Stanczyk F.Z. (2008). Progesterone receptors: Form and function in brain. Front. Neuroendocr..

[17] Morriss M.C., Zimmerman R.A., Bilaniuk L.T., Hunter J.V., Haselgrove J.C. (1999). Changes in brain water diffusion during childhood. Neuroradiology.

[18] Taveggia C., Feltri M.L., Wrabetz L. (2010). Signals to promote myelin formation and repair. Nat. Rev. Neurol..

[19] Rupprecht R., Berning B., Hauser C.A., Holsboer F., Reul J.M. (1996). Steroid receptor-mediated effects of neuroactive steroids: Characterization of structure-activity relationship. Eur. J. Pharmacol..

[20] Bitran D., Shiekh M., McLeod M. (1995). Anxiolytic Effect of Progesterone is Mediated by the Neurosteroid Allopregnanolone at Brain GABA A Receptors. J. Neuroendocrinol..

[21] Smith S.S. (2002). Withdrawal properties of a neuroactive steroid: Implications for GABAA receptor gene regulation in the brain and anxiety behavior. Steroids.

[22] Ströhle A., Romeo E., Hermann B., Pasini A., Spalletta G., Di Michele F., Holsboer F., Rupprecht R. (1999). Concentrations of 3α-reduced neuroactive steroids and their precursors in plasma of patients with major depression and after clinical recovery. Biol. Psychiatry.

[23] Yen J.-Y., Chang S.-J., Long C.-Y., Tang T.-C., Chen C.-C., Yen C.-F. (2012). Working memory deficit in premenstrual dysphoric disorder and its associations with difficulty in concentrating and irritability. Compr. Psychiatry.

[24] Zheng P. (2009). Neuroactive steroid regulation of neurotransmitter release in the CNS: Action, mechanism and possible significance. Prog. Neurobiol..

[25] Stárka L., Dušková M., Hill M. (2015). Dehydroepiandrosterone: A neuroactive steroid. J. Steroid. Biochem. Mol. Biol..

[26] Quinn T., Greaves R., Badoer E., Walker D. (2018). DHEA in Prenatal and Postnatal Life: Implications for Brain and Behavior. Vitam. Horm..

[27] Sullivan Mitchell E., Fugate Woods N. (2001). Midlife women’s attributions about perceived memory changes: Observations from the Seattle Midlife Women’s Health Study. J. Womens Health Gend Based Med..

[28] Rocca W.A., Mielke M.M., Vemuri P., Miller V.M. (2014). Sex and gender differences in the causes of dementia: A narrative review. Maturitas.

[29] Rentz D.M., Weiss B.K., Jacobs E.G., Cherkerzian S., Klibanski A., Remington A., Aizley H., Goldstein J.M. (2017). Sex differences in episodic memory in early midlife: Impact of reproductive aging. Menopause.

[30] Lui L.-Y., Stone K., Cauley J.A., Hillier T., Yaffe K. (2003). Bone loss predicts subsequent cognitive decline in older women: The study of osteoporotic fractures. J. Am. Geriatr. Soc..

[31] Barrett-Connor E., Goodman-Gruen D. (1999). Cognitive Function and Endogenous Sex Hormones in Older Women. J. Am. Geriatr. Soc..

[32] Fuh J.-L., Wang S.-J., Lee S.-J., Lu S.-R., Juang K.-D. (2006). A longitudinal study of cognition change during early menopausal transition in a rural community. Maturitas.

[33] Greendale G.A., Huang M.-H., Wight R.G., Seeman T., Luetters C., Avis N.E., Johnston J., Karlamangla A.S. (2009). Effects of the menopause transition and hormone use on cognitive performance in midlife women. Neurology.

[34] Kuh D., Cooper R., Moore A., Richards M., Hardy R. (2018). Age at menopause and lifetime cognition: Findings from a British birth cohort study. Neurology.

[35] Geerlings M.I., Ruitenberg A., Witteman J.C.M., Van Swieten J.C., Hofman A., Van Duijn C.M., Breteler M.M.B., Launer L.J. (2001). Reproductive Period and Risk of Dementia in Postmenopausal Women. JAMA.

[36] Tang M.-X., Jacobs D., Stern Y., Marder K., Schofield P., Gurland B., Andrews H., Mayeux R. (1996). Effect of oestrogen during menopause on risk and age at onset of Alzheimer’s disease. Lancet.

[37] Waring S.C., Rocca W.A., Petersen R.C., O’Brien P.C., Tangalos E.G., Kokmen E. (1999). Postmenopausal estrogen replacement therapy and risk of AD: A population-based study. Neurology.

[38] Zandi P.P., Carlson M.C., Plassman B.L., Welsh-Bohmer K.A., Mayer L.S., Steffens D.C., Breitner J.C.S. (2002). For the Cache County Memory Study Investigators Hormone Replacement Therapy and Incidence of Alzheimer Disease in Older Women the Cache County Study. JAMA.

[39] Shao H., Breitner J.C., Whitmer R.A., Wang J., Hayden K., Wengreen H., Corcoran C., Tschanz J., Norton M., Munger R. (2012). Hormone therapy and Alzheimer disease dementia: New findings from the Cache County Study. Neurology.

[40] Imtiaz B., Taipale H., Tanskanen A., Tiihonen M., Kivipelto M., Heikkinen A.-M., Tiihonen J., Soininen H., Hartikainen S., Tolppanen A.-M. (2017). Risk of Alzheimer’s disease among users of postmenopausal hormone therapy: A nationwide case-control study. Maturitas.

[41] Yaffe K., Sawaya G., Lieberburg I., Grady D. (1998). Estrogen therapy in postmenopausal women: Effects on cognitive function and dementia. JAMA.

[42] Rapp S.R., Espeland M.A., Shumaker S.A., Henderson V.W., Brunner R.L., Manson J.E., Gass M.L., Stefanick M.L., Lane D.S., Hays J. (2003). Effect of estrogen plus progestin on global cognitive function in postmenopausal women: The Women’s Health Initiative Memory Study: A randomized controlled trial. JAMA.

[43] Espeland M.A., Rapp S.R., Shumaker S.A., Brunner R., Manson J.E., Sherwin B.B., Hsia J., Margolis K.L., Hogan P.E., Wallace R. (2004). Conjugated Equine Estrogens and Global Cognitive Function in Postmenopausal Women: Women??? Health Initiative Memory Study. Obstet. Gynecol. Surv..

[44] Kang J.H., Weuve J., Grodstein F. (2004). Postmenopausal hormone therapy and risk of cognitive decline in community-dwelling aging women. Neurology.

[45] Kang J.H., Grodstein F. (2012). Postmenopausal hormone therapy, timing of initiation, APOE and cognitive decline. Neurobiol. Aging.

[46] Lethaby A., Hogervorst E., Richards M., Yesufu A., Yaffe K. (2008). Hormone replacement therapy for cognitive function in postmenopausal women. Cochrane Database Syst. Rev..

[47] Coker L.H., Espeland M.A., Rapp S.R., Legault C., Resnick S.M., Hogan P., Gaussoin S., Dailey M., Shumaker S.A. (2010). Postmenopausal hormone therapy and cognitive outcomes: The Women’s Health Initiative Memory Study (WHIMS). J. Steroid Biochem. Mol. Biol..

[48] Marder K., Sano M. (2000). Estrogen to treat Alzheimer’s disease: Too little, too late? So what’s a woman to do?. Neurology.

[49] Whitmer R.A., Quesenberry C.P., Zhou J., Yaffe K. (2011). Timing of hormone therapy and dementia: The critical window theory revisited. Ann. Neurol..

[50] Henderson V.W., Benke K.S., Green R.C., Cupples L.A., Farrer L.A., MIRAGE Study Group (2005). Postmenopausal hormone therapy and Alzheimer’s disease risk: Interaction with age. J. Neurol Neurosurg Psychiatry.

[51] Petitti D.B., Crooks V.C., Chiu V., Buckwalter J.G., Chui H.C. (2008). Incidence of dementia in long-term hormone users. Am. J. Epidemiol..

[52] Ryan J., Carrière I., Scali J., Dartigues J.-F., Tzourio C., Poncet M., Ritchie K., Ancelin M.-L. (2009). Characteristics of hormone therapy, cognitive function, and dementia: The prospective 3C Study. Neurology.

[53] Espeland M.A., Shumaker S.A., Leng I., Manson J.E., Brown C.M., Leblanc E.S., Vaughan L., Robinson J., Rapp S.R., Goveas J.S. (2013). Long-term effects on cognitive function of postmenopausal hormone therapy prescribed to women aged 50 to 55 years. JAMA Intern. Med..

[54] Savolainen-Peltonen H., Rahkola-Soisalo P., Hoti F., Vattulainen P., Gissler M., Ylikorkala O., Mikkola T.S. (2019). Use of postmenopausal hormone therapy and risk of Alzheimer’s disease in Finland: Nationwide case-control study. BMJ.

[55] Alvarez-De-La-Rosa M., Silva I., Nilsen J., Pérez M.M., García-Segura L.M., Ávila J., Naftolin F. (2005). Estradiol Prevents Neural Tau Hyperphosphorylation Characteristic of Alzheimer’s Disease. Ann. N. Y. Acad. Sci..

[56] Yue X., Lu M., Lancaster T., Cao P., Honda S.-I., Staufenbiel M., Harada N., Zhong Z., Shen Y., Li R. (2005). Brain estrogen deficiency accelerates A plaque formation in an Alzheimer’s disease animal model. Proc. Natl. Acad. Sci. USA.

[57] Thomas T., Bryant M., Clark L., Garces A., Rhodin J. (2001). Estrogen and Raloxifene Activities on Amyloid-β-Induced Inflammatory Reaction. Microvasc. Res..

[58] Leblanc E.S., Janowsky J., Chan B.K.S., Nelson H.D. (2001). Hormone Replacement Therapy and Cognition: Systematic Review and Meta-Analysis. Obstet. Gynecol. Surv..

[59] O’Brien J., Jackson J.W., Grodstein F., Blacker D., Weuve J. (2014). Postmenopausal hormone therapy is not associated with risk of all-cause dementia and Alzheimer’s disease. Epidemiol. Rev..

[60] Kantarci K., Lowe V.J., Lesnick T.G., Tosakulwong N., Bailey K.R., Fields J.A., Shuster L.T., Zuk S.M., Senjem M.L., Mielke M.M. (2016). Early Postmenopausal Transdermal 17β-Estradiol Therapy and Amyloid-β Deposition. J. Alzheimer’s Dis..

[61] Mulnard R.A., Cotman C.W., Kawas C., Van Dyck C.H., Sano M., Doody R., Koss E., Pfeiffer E., Jin S., Gamst A. (2000). Estrogen Replacement Therapy for Treatment of Mild to Moderate Alzheimer Disease: A Randomized Controlled Trial. Obstet. Gynecol. Surv..

[62] Hogervorst E. (2013). Estrogen and the brain: Does estrogen treatment improve cognitive function?. Menopause Int..

[63] Guérin E., Prud’Homme D., Goldfield G. (2017). Trajectories of mood and stress and relationships with protective factors during the transition to menopause: Results using latent class growth modeling in a Canadian cohort. Arch. Women’s Ment. Health.

[64] Soares C.N. (2019). Depression and Menopause: An Update on Current Knowledge and Clinical Management for this Critical Window. Med. Clin. N. Am..

[65] Bruyneel M. (2015). Sleep disturbances in menopausal women: Aetiology and practical aspects. Maturitas.

[66] Bernstein M., Rouget P., Archinard M., Morabia A. (1998). Body weight preoccupation in middle-age and ageing women: A general population survey. Int. J. Eat. Disord..

[67] Mouton C., Reame N., Salamone L., Stellato R., Gold E.B., Sternfeld B., Kelsey J.L., Brown C. (2000). Relation of Demographic and Lifestyle Factors to Symptoms in a Multi-Racial/Ethnic Population of Women 40–55 Years of Age. Am. J. Epidemiol..

[68] Sowislo J.F., Orth U. (2013). Does low self-esteem predict depression and anxiety? A meta-analysis of longitudinal studies. Psychol. Bull..

[69] Hunter M., Rendall M. (2007). Bio-psycho-socio-cultural perspectives on menopause. Best Pract. Res. Clin. Obstet. Gynaecol..

[70] Darwish M., Atlantis E., Mohamed-Taysir T. (2014). Psychological outcomes after hysterectomy for benign conditions: A systematic review and meta-analysis. Eur. J. Obstet. Gynecol. Reprod. Biol..

[71] Gibson C.J., Joffe H., Bromberger J.T., Thurston R.C., Lewis T.T., Khalil N., Matthews K.A. (2012). Mood Symptoms After Natural Menopause and Hysterectomy with and Without Bilateral Oophorectomy Among Women in Midlife. Obstet. Gynecol..

[72] Rocca W.A., Grossardt B.R., Geda Y.E., Gostout B.S., Bower J.H., Maraganore D.M., de Andrade M., Melton L.J. (2008). Long-term risk of depressive and anxiety symptoms after early bilateral oophorectomy. Menopause.

[73] Chou P.-H., Lin C.-H., Cheng C., Chang C.-L., Tsai C.-J., Tsai C.-P., Lan T.-H., Chan C.-H. (2015). Risk of depressive disorders in women undergoing hysterectomy: A population-based follow-up study. J. Psychiatr. Res..

[74] Wilson L., Pandeya N., Byles J., Mishra G. (2018). Hysterectomy and incidence of depressive symptoms in midlife women: The Australian Longitudinal Study on Women’s Health. Epidemiol. Psychiatr. Sci..

[75] Schmidt P.J., Ben Dor R., Martinez P.E., Guerrieri G.M., Harsh V.L., Thompson K., Koziol D.E., Nieman L.K., Rubinow D.R. (2015). Effects of Estradiol Withdrawal on Mood in Women with Past Perimenopausal Depression: A Randomized Clinical Trial. JAMA Psychiatry.

[76] Rubinow D.R., Johnson S.L., Schmidt P.J., Girdler S., Gaynes B. (2015). Efficacy of Estradiol in Perimenopausal Depression: So Much Promise and So Few Answers: Research Article: Efficacy of Estradiol in Perimenopausal Depression. Depress. Anxiety.

[77] Bethea C.L., Mirkes S.J., Su A., Michelson D. (2002). Effects of oral estrogen, raloxifene and arzoxifene on gene expression in serotonin neurons of macaques. Psychoneuroendocrinology.

[78] Pau K.Y., Hess D.L., Kohama S., Bao J., Pau C.Y., Spies H.G. (2000). Oestrogen upregulates noradrenaline release in the mediobasal hypothalamus and tyrosine hydroxylase gene expression in the brainstem of ovariectomized rhesus macaques. J. Neuroendocr..

[79] Maki P.M., Kornstein S.G., Joffe H., Bromberger J.T., Freeman E.W., Athappilly G., Bobo W.V., Rubin L.H., Koleva H.K., Cohen L.S. (2018). Guidelines for the evaluation and treatment of perimenopausal depression: Summary and recommendations. Menopause.

[80] Bromberger J.T., Kravitz H.M., Youk A., Schott L.L., Joffe H. (2016). Patterns of depressive disorders across 13 years and their determinants among midlife women: SWAN mental health study. J. Affect. Disord..

[81] Cohen L.S., Soares C.N., Vitonis A.F., Otto M.W., Harlow B.L. (2006). Risk for New Onset of Depression During the Menopausal Transition: The Harvard Study of Moods and Cycles. Arch Gen. Psychiatry.

[82] Hickey M., Schoenaker D.A.J.M., Joffe H., Mishra G.D. (2016). Depressive symptoms across the menopause transition: Findings from a large population-based cohort study. Menopause.

[83] Woods N.F., Smith-DiJulio K., Percival D.B., Tao E.Y., Mariella A., Mitchell E.S. (2008). Depressed mood during the menopausal transition and early postmenopause: Observations from the Seattle Midlife Women’s Health Study. Menopause.

[84] Freeman E.W., Sammel M.D., Boorman D.W., Zhang R. (2014). Longitudinal pattern of depressive symptoms around natural menopause. JAMA Psychiatry.

[85] Gordon J.L., Rubinow D.R., Eisenlohr-Moul T.A., Leserman J., Girdler S.S. (2016). Estradiol variability, stressful life events, and the emergence of depressive symptomatology during the menopausal transition. Menopause.

[86] Khoo S.K., Coglan M., Battistutta D., Tippett V., Raphael B. (1998). Hormonal treatment and psychological function during the menopausal transition: An evaluation of the effects of conjugated estrogens/cyclic medroxyprogesterone acetate. Climacteric.

[87] Santoro N., Teal S., Gavito C., Cano S., Chosich J., Sheeder J. (2015). Use of a levonorgestrel-containing intrauterine system with supplemental estrogen improves symptoms in perimenopausal women: A pilot study. Menopause.

[88] Haines C.J., Yim S.F., Chung T.K., Lam C.W., Lau E.W., Ng M.H., Chin R., Lee D.T. (2003). A prospective, randomized, placebo-controlled study of the dose effect of oral oestradiol on menopausal symptoms, psychological well being, and quality of life in postmenopausal Chinese women. Maturitas.

[89] Girdler S.S., O’Briant C., Steege J., Grewen K., Light K.C. (1999). A Comparison of the Effect of Estrogen with or without Progesterone on Mood and Physical Symptoms in Postmenopausal Women. J. Women’s Health Gender Based Med..

[90] Almeida O.P., Lautenschlager N.T., Vasikaran S., Leedman P., Gelavis A., Flicker L. (2006). A 20-week randomized controlled trial of estradiol replacement therapy for women aged 70 years and older: Effect on mood, cognition and quality of life. Neurobiol. Aging.

[91] Yalamanchili V., Gallagher J.C. (2012). Treatment with hormone therapy and calcitriol did not affect depression in older postmenopausal women: No interaction with estrogen and vitamin D receptor genotype polymorphisms. Menopause.

[92] Gleason C.E., Dowling N.M., Wharton W., Manson J.E., Miller V.M., Atwood C.S., Brinton E.A., Cedars M.I., Lobo R.A., Merriam G.R. (2015). Effects of Hormone Therapy on Cognition and Mood in Recently Postmenopausal Women: Findings from the Randomized, Controlled KEEPS–Cognitive and Affective Study. PLoS Med..

[93] Schmidt P.J., Nieman L., Danaceau M.A., Tobin M.B., Roca C.A., Murphy J.H., Rubinow D.R. (2000). Estrogen replacement in perimenopause-related depression: A preliminary report. Am. J. Obstet. Gynecol..

[94] Soares C.N., Almeida O.P., Joffe H., Cohen L.S. (2001). Efficacy of estradiol for the treatment of depressive disorders in perimenopausal women: A double-blind, randomized, placebo-controlled trial. Arch. Gen. Psychiatry.

[95] Rudolph I., Palombo-Kinne E., Kirsch B., Mellinger U., Breitbarth H., Gräser T. (2004). Influence of a continuous combined HRT (2 mg estradiol valerate and 2 mg dienogest) on postmenopausal depression. Climacteric.

[96] Morrison M.F., Kallan M.J., Have T.T., Katz I., Tweedy K., Battistini M. (2004). Lack of efficacy of estradiol for depression in postmenopausal women: A randomized, controlled trial. Biol. Psychiatry.

[97] Joffe H., Petrillo L.F., Koukopoulos A., Viguera A.C., Hirschberg A., Nonacs R., Somley B., Pasciullo E., White D.P., Hall J.E. (2011). Increased estradiol and improved sleep, but not hot flashes, predict enhanced mood during the menopausal transition. J. Clin. Endocrinol. Metab..

[98] Green S.M., Key B.L., McCabe R.E. (2015). Cognitive-behavioral, behavioral, and mindfulness-based therapies for menopausal depression: A review. Maturitas.

[99] Kravitz H.M., Ganz P.A., Bromberger J., Powell L.H., Sutton-Tyrrell K., Meyer P.M. (2003). Sleep difficulty in women at midlife: A community survey of sleep and the menopausal transition. Menopause.

[100] Luyster F.S., Strollo P.J., Zee P.C., Walsh J.K. (2012). Sleep: A Health Imperative. Sleep.

[101] Baker F.C., De Zambotti M., Colrain I.M., Bei B. (2018). Sleep problems during the menopausal transition: Prevalence, impact, and management challenges. Nat. Sci. Sleep.

[102] Kravitz H.M., Joffe H. (2011). Sleep during the perimenopause: A SWAN story. Obstet. Gynecol. Clin. N. Am..

[103] Sowers M.F., Zheng H., Kravitz H.M., Matthews K., Bromberger J.T., Gold E.B., Owens J., Consens F., Hall M. (2008). Sex Steroid Hormone Profiles are Related to Sleep Measures from Polysomnography and the Pittsburgh Sleep Quality Index. Sleep.

[104] Gallicchio L., Whiteman M.K., Tomic D., Miller K.P., Langenberg P., Flaws J.A. (2006). Type of menopause, patterns of hormone therapy use, and hot flashes. Fertil. Steril..

[105] Archer D.F., Sturdee D.W., Baber R., De Villiers T.J., Pines A., Freedman R.R., Gompel A., Hickey M., Hunter M.S., Lobo R.A. (2011). Menopausal hot flushes and night sweats: Where are we now?. Climacteric.

[106] Rance N.E., Dacks P.A., Mittelman-Smith M.A., Romanovsky A.A., Krajewski-Hall S.J. (2013). Modulation of body temperature and LH secretion by hypothalamic KNDy (kisspeptin, neurokinin B and dynorphin) neurons: A novel hypothesis on the mechanism of hot flushes. Front. Neuroendocr..

[107] Jehan S., Masters-Isarilov A., Salifu I., Zizi F., Jean-Louis G., Pandi-Perumal S.R., Gupta R., Brzezinski A., McFarlane S.I. (2015). Sleep Disorders in Postmenopausal Women. J. Sleep Disord. Ther..

[108] Kimura M. (2005). Minireview: Gender-specific sleep regulation. Sleep Biol. Rhythm..

[109] Feinberg I. (1974). Changes in sleep cycle patterns with age. J. Psychiatr. Res..

[110] Viola-Saltzman M., Watson N.F., Bogart A., Goldberg J., Buchwald D. (2010). High Prevalence of Restless Legs Syndrome among Patients with Fibromyalgia: A Controlled Cross-Sectional Study. J. Clin. Sleep Med..

[111] Attarian H.P., Viola-Saltzman M. (2013). Sleep Disorders in Women.

[112] Gupta R., Goel D., Ahmed S., Dhar M., Lahan V. (2014). What patients do to counteract the symptoms of Willis-Ekbom disease (RLS/WED): Effect of gender and severity of illness. Ann. Indian Acad. Neurol..

[113] Manconi M., Ulfberg J., Berger K., Ghorayeb I., Wesström J., Fulda S., Allen R.P., Pollmächer T. (2012). When gender matters: Restless legs syndrome. Report of the “RLS and woman” workshop endorsed by the European RLS Study Group. Sleep Med. Rev..

[114] Dancey D.R., Hanly P.J., Soong C., Lee B., Hoffstein V. (2001). Impact of Menopause on the Prevalence and Severity of Sleep Apnea. Chest.

[115] Resta O., Bonfitto P., Sabato R., De Pergola G., Barbaro M.P.F. (2004). Prevalence of obstructive sleep apnoea in a sample of obese women: Effect of menopause. Diabetes Nutr. Metab..

[116] MacLennan A.H., Broadbent J.L., Lester S., Moore V. (2004). Oral oestrogen and combined oestrogen/progestogen therapy versus placebo for hot flushes. Cochrane Database Syst. Rev..

[117] Cintron D., Lipford M., Larrea-Mantilla L., Spencer-Bonilla G., Lloyd R., Gionfriddo M.R., Gunjal S., Farrell A.M., Miller V.M., Murad M.H. (2017). Efficacy of menopausal hormone therapy on sleep quality: Systematic review and meta-analysis. Endocrine.

[118] Polo-Kantola P., Erkkola R., Helenius H., Irjala K., Polo O. (1998). When does estrogen replacement therapy improve sleep quality?. Am. J. Obstet. Gynecol..

[119] Savolainen-Peltonen H., Hautamäki H., Tuomikoski P., Ylikorkala O., Mikkola T.S. (2014). Health-related quality of life in women with or without hot flashes: A randomized placebo-controlled trial with hormone therapy. Menopause.

[120] Welton A.J., Vickers M.R., Kim J., Ford D.A., Lawton B., MacLennan A.H., Meredith S.K., Martin J., Meade T.W. (2008). Health related quality of life after combined hormone replacement therapy: Randomised controlled trial. BMJ.

[121] Attarian H., Hachul H., Guttuso T., Phillips B. (2015). Treatment of chronic insomnia disorder in menopause: Evaluation of literature. Menopause.

[122] Schüssler P., Kluge M., Yassouridis A., Dresler M., Held K., Zihl J., Steiger A. (2008). Progesterone reduces wakefulness in sleep EEG and has no effect on cognition in healthy postmenopausal women. Psychoneuroendocrinology.

